# P28 Multi-system and cavernous sinus inflammatory syndrome presenting with evolving multiple cranial neuropathies following zoledronate infusion

**DOI:** 10.1093/rap/rkab068.027

**Published:** 2021-10-19

**Authors:** Tavishi Kanwar, Clare Thornton, Lucy Childs, Tasanee Braithwaite

**Affiliations:** 1 Kingston Hospital Foundation Trust, London, United Kingdom; 2 University College Hospital London NHS Foundation Trust, London, United Kingdom; 3 Guy’s and St Thomas’ NHS Foundation Trust, London, United Kingdom; 4 Medical Eye Unit, Guy’s and St Thomas’ NHS Foundation Trust, London, United Kingdom

## Abstract

**Case report - Introduction:**

Bisphosphonates are known to rarely cause multi-system inflammation, including multiple cranial neuropathies. This is possibly via provoking transient cytokine storm. The literature reports bisphosphonate-associated orbital inflammatory syndrome, and one case of retrobulbar optic neuritis following zoledronate. Bisphosphonate manufacturers report conjunctivitis, blurred vision, scleritis, orbital inflammation, uveitis and episcleritis as ocular side effects. Separately, neurological sequalae, including cranial neuropathies, are reported following COVID-19 infection and vaccination. Here, we report the first case of cavernous sinus inflammation temporally related to both zoledronate infusion, and more remotely, to Pfizer-BioNTech COVID-19 vaccination.

**Case report - Case description:**

A 76-year-old white man developed fever, bony leg pain – which rendered him unable to walk – and frontal headache, within 8 hours of his first zoledronate infusion for osteoporosis. A few weeks earlier he received his first Pfizer-BioNTech COVID-19 vaccine. His General Practitioner commenced a short course of low-dose oral prednisolone for the episode.

One week later, off prednisolone, the headache localised around the left eye. He developed horizontal diplopia associated with abduction deficit. He was diagnosed with left VIth nerve palsy. He was started on high-dose steroids and clopidogrel (with PPI) with neuroimaging to exclude stroke or venous sinus thrombosis. Two weeks later, the diplopia worsened over 4 days, with new left adduction deficit (-2 limitation), left ptosis 1-2mm and anisocoria 0.5-1mm R>L suggestive of partial third nerve palsy and early Horner’s syndrome. Ocular and neurological examinations were otherwise normal.

He wore varifocals and had migraines, osteoporosis, and asthma, for which he used inhalers. He worked in visual arts and was an ex-smoker (>50 years) with moderate alcohol intake.

Blood results revealed CRP 38mg/L, but otherwise normal inflammation/vasculitis/infection screen; anti-thyroglobulin antibodies were >4000 U/ml; GQ1P, Creatinine Kinase, anti-ganglioside, and Anti-AChR/MuSK antibodies were normal. CT head and Optical Coherence Tomography were unremarkable. An enhanced MRI of the brain and orbits revealed abnormal thickening and T2 hyper-intensity of the left oculomotor nerve, most notably involving the left canalicular portion. The left cavernous sinus also appeared asymmetrically bulky with a rind of abnormal enhancing soft tissue in the left cavernous sinus. Subtle STIR hyper-intensity was also observed in the ipsilateral CN III-innervated extra-ocular muscles.

After a 6-week course of tapering prednisolone, the vertical diplopia and leg swelling persisted; the horizontal diplopia and headaches had resolved. By 3 months, there was resolution with mild residual visual changes.

**Case report - Discussion:**

We report a constellation of symptoms relating to multi-system inflammatory syndrome involving the cavernous sinus.

There is a lack of epidemiological data on the incidence of this rare presentation in the population. This case has close temporal association to bisphosphonate infusion (<12h) and weaker association to coronavirus vaccination (<3wk). It is difficult to determine whether this is a rare presentation of a known drug reaction, a more delayed presentation of a vaccine reaction or whether these events were coincidental. A further possibility in this case is a combined predisposition resulting from both vaccination and bisphosphonate infusion.

This case highlights a wider issue relating to the challenging possibility of ascertainment bias and increased ‘Yellow Card’ reporting of rare presentations during this historic global coronavirus pandemic, which may or may not have any true causal association to vaccination. There is difficulty in disentangling a true vaccine reaction from an unrelated presentation of a rare condition with an unknown baseline incidence rate. This is especially topical given that the majority of the population are receiving the coronavirus vaccination at this time. We also question what a plausible cut-off point would be to propose a temporal relationship for an adverse reaction; in the literature, adverse reactions have been postulated to develop beyond 1 month after the provoking agent.

**Case report - Key learning points:**

- This case highlights the need for urgent assessment, investigations including neurological imaging and consultant input in patients with evolving cranial neuropathy. The priority is to rule out thrombotic, compressive, inflammatory and infectious pathology in the cavernous sinus, venous sinus, orbit and orbital apex.
- Pathology of the cavernous sinus presents with variable involvement of CN III, IV, V and VI and Horner’s syndrome. A differential for this case would be superior orbital fissure syndrome, which also presents with multiple oculomotor cranial neuropathies; it involves these cranial nerves and the ophthalmic branch of CN V. Orbital apex syndrome is SOF with a loss of vision due to additional CNII involvement.
- The neuro-radiology differential included inflammatory, infiltrative, granulomatous and neoplastic aetiologies and that there was sufficient existing evidence to exclude brainstem pathology.
- Through communication between specialties, the temporal relationship was established, and clinical examination and extensive investigation further honed the differential to either inflammatory or vascular. Since it was temporally related to the zolendronate infusion, it seemed plausible it was related. We demonstrate the need for multi-disciplinary collaboration for these patients between rheumatology, ophthalmology and neuro-radiology.

